# The effect of lysergic acid diethylamide (LSD) on whole-brain functional and effective connectivity

**DOI:** 10.1038/s41386-023-01574-8

**Published:** 2023-04-25

**Authors:** Peter Bedford, Daniel J. Hauke, Zheng Wang, Volker Roth, Monika Nagy-Huber, Friederike Holze, Laura Ley, Patrick Vizeli, Matthias E. Liechti, Stefan Borgwardt, Felix Müller, Andreea O. Diaconescu

**Affiliations:** 1 Krembil Centre for Neuroinformatics, Centre for Addiction and Mental Health (CAMH), Toronto, ON Canada; 2 Centre for Medical Image Computing, Department of Computer Science, University College London, London, UK; 3 Department of Mathematics and Computer Science, University of Basel, Basel, Switzerland; 4 Division of Clinical Pharmacology and Toxicology, Department of Biomedicine and Department of Clinical Research, University Hospital Basel, University of Basel, Basel, Switzerland; 5 Department of Psychiatry and Psychotherapy, Translational Psychiatry, Lübeck, Germany; 6 Department of Psychiatry (UPK), University of Basel, Basel, Switzerland; 7 Department of Psychiatry, University of Toronto, Toronto, ON Canada; 8 Institute of Medical Sciences, University of Toronto, Toronto, ON Canada; 9 Department of Psychology, University of Toronto, Toronto, ON Canada

**Keywords:** Diagnostic markers, Consciousness, Neurotransmitters

## Abstract

Psychedelics have emerged as promising candidate treatments for various psychiatric conditions, and given their clinical potential, there is a need to identify biomarkers that underlie their effects. Here, we investigate the neural mechanisms of lysergic acid diethylamide (LSD) using regression dynamic causal modelling (rDCM), a novel technique that assesses whole-brain effective connectivity (EC) during resting-state functional magnetic resonance imaging (fMRI). We modelled data from two randomised, placebo-controlled, double-blind, cross-over trials, in which 45 participants were administered 100 μg LSD and placebo in two resting-state fMRI sessions. We compared EC against whole-brain functional connectivity (FC) using classical statistics and machine learning methods. Multivariate analyses of EC parameters revealed predominantly stronger interregional connectivity and reduced self-inhibition under LSD compared to placebo, with the notable exception of weakened interregional connectivity and increased self-inhibition in occipital brain regions as well as subcortical regions. Together, these findings suggests that LSD perturbs the Excitation/Inhibition balance of the brain. Notably, whole-brain EC did not only provide additional mechanistic insight into the effects of LSD on the Excitation/Inhibition balance of the brain, but EC also correlated with global subjective effects of LSD and discriminated experimental conditions in a machine learning-based analysis with high accuracy (91.11%), highlighting the potential of using whole-brain EC to decode or predict subjective effects of LSD in the future.

## Introduction

Psychedelics like psilocybin and lysergic acid diethylamide (LSD) have emerged as promising new treatment candidates for a variety of psychiatric conditions including substance dependence [1–3], major depression [4], anxiety disorders [5], and adjustment disorders [6–8]. Since both LSD and psilocybin primarily act on 5-HT_2A_ receptors, an increasing number of clinical trials using psychedelics have been registered to evaluate their efficacy for treatment of depressive disorders and other psychiatric conditions [9]. Given this clinical potential and growing interest in precision psychiatry, we sought to examine the neural mechanisms that underpin whole-brain effects of LSD by employing computational modelling and machine learning.

Functional connectivity (FC) [10–18] and effective connectivity (EC) [19, 20] have already shown promise in providing a framework for uncovering the neural mechanisms underlying LSD. Both connectivity measures are widely used, but are interpretatively distinct: FC is commonly assessed using Pearson correlation coefficients—a measure of the linear relationship—between the BOLD signal time series of two distinct brain regions. In a general linear model, the squared correlation coefficient represents the proportion of one signal’s variance, which can be explained by another signal, and vice versa. Though much of the research investigating FC changes under LSD suggests that FC is a promising candidate for a clinically-relevant biomarker [10–18], FC can be limited in terms of its interpretability.

Firstly, FC is an undirected measure of connectivity, because computation of the correlation coefficient is commutative. Secondly, it ignores two important organisational principles of the cortex: (1) the asymmetry of connections [21, 22] and (2) the presence of gain regulation within a cortical region or ’self-connections’. In contrast to FC, EC rests on a mechanistic model of how the data were generated [23] and estimates both, asymmetry and the gain within a cortical region. Here, we adopt a mechanistic perspective and examine LSD-driven changes in *directed* influences between nodes, as well as asymmetry and self-inhibition across the brain.

In this exploratory analysis, we estimated whole-brain EC using regression dynamic causal modelling (rDCM; [24]), a recently developed variant of dynamic causal modelling (DCM; [25]). rDCM allows estimation of whole-brain EC by applying several modifications and simplifications to the original DCM framework by reformulating a linear DCM in the time domain as a linear Bayesian regression in the frequency domain [26]. Furthermore, this model has been recently extended to allow modelling of task-free or resting-state magnetic resonance imaging (rs-MRI) data [24, 27], enabling us to study—for the first time—how whole-brain effective connectivity is altered under LSD. Modelling asymmetry of directed influences and within-region gain can potentially provide additional useful information about the neural mechanisms underlying LSD effects.

To assess this, we also compared FC and EC in terms of their ability to distinguish LSD from placebo at the individual level. In future studies, these computationally-informed biomarkers could potentially be leveraged to predict the subjective effects of psychedelics.

## Materials and methods

### Participants

Data from two randomised, placebo-controlled, double-blind, cross-over trials were aggregated comprising 20 healthy participants (10 male, 10 female; age 32 ± 11 [mean ± SD]; range 25 − 60 years; body weight, 68.8 ± 7.7 kg; trial A: NCT02308969 [28, 29]) and 25 healthy participants (12 men, 13 women; age 28 ± 4.3; range 25 − 45 years; body weight, 70.2 ± 11.2 kg; trial B: NCT03019822 [30]). More details including previous use of hallucinogens and in- and exclusion criteria are provided in the Supplement. Both studies were approved by the Ethics Committee for Northwest/Central Switzerland and by the Federal Office of Public Health. All participants provided written consent and received monetary compensation.

### Experimental procedure

Participants were administered 100 μg LSD orally in capsules (trial A) or vials (trial (B) and identical mannitol and ethanol-filled placebo capsules/vials in a cross-over design across two separate experimental sessions with a time between sessions of at least 7 days (17 ± 35.3 days [median ± SD], range: 7−182 days). Each session included an assessment of brain activity during rest using fMRI, which was acquired 140.5 ± 10.9 min [median ± SD] (range: 121−200 min) after administration of LSD or placebo. (see Supplement for data acquisition parameters and preprocessing procedure). Participants were instructed to close their eyes and remain awake during the scan. Subjective effects were assessed with the 5 Dimensions of Altered States of Consciousness (5D-ASC) scale [31, 32] 11h after drug administration. Participants were asked to retrospectively rate the drug effects.

### Data analysis

#### Functional connectivity

We computed FC using Pearson correlations between the BOLD signal time series of each pair of the 132 distinct brain regions derived from the Harvard-Oxford atlas, yielding 8648 unique correlation coefficients. Please, see Table S1 in the Supplement for details on software for all subsequent analyses.

#### Effective connectivity

EC for fully connected whole-brain networks was estimated from the raw time series of all 132 regions of interest (ROIs) using rDCM [24, 26, 27] (Supplement).

#### Statistical analysis

Connectivity changes under LSD were investigated using two complementary approaches: (1) linear multivariate tests and (2) machine learning, which allowed us to assess multi-variate, non-linear changes and quantify how well we were able to distinguish LSD from placebo at the single-participant level. This second analysis served as an additional test to gauge the potential of using connectivity features to decode or predict subjective effects in future studies. We supplement these results with mass-univariate (paired-sample) t-tests with Benjamini and Hochberg [33] correction for multiple testing to control the false discovery rate (FDR; *α* < 0.05).

*Partial least squares correlation analysis*. Mean-centred task or behavioural partial least squares correlation (PLSC) analysis was used to capture FC or EC that maximally represented differences between LSD and placebo conditions or that maximally correlated with subjective effects (5D-ASC global score), respectively [34]. A detailed investigation of subjective effects (including subscales and pleasant vs unpleasant effects) is beyond the scope of this paper and will be investigated in future studies. Singular value decomposition (SVD) was applied to the mean-centred matrix of FC, EC, and self-connections separately. SVD re-expresses this matrix as a set of orthogonal singular vectors or latent variables (LVs), the number of which is equivalent to the total number of conditions for mean-centred task PLSC and one LV for the behavioral PLSC. The LVs can be understood analogous to principal components in principal component analsis and account for the covariance of the original mean-centred matrix in decreasing order of magnitude. Statistical significance of LVs and reliability of region loadings on the LVs were assessed using permutation tests with 2000 permutations and bootstrapping with 2000 samples, respectively. To account for inter-individual variability in baseline physiology (Table S2), connectivity features were first explained by baseline heart rate, diastolic and systolic blood pressure, and body temperature and PLSC was performed on the residuals that could not be explained by physiological effects (Supplement).

*Machine learning analysis*. We trained random forest classifiers [35] on either whole-brain FC or EC as features to classify conditions (LSD versus placebo; Supplement, Fig. S1). Feature preprocessing consisted of pruning and covariate correction for physiological variables (Supplement) and was embedded in a 5-fold cross-validation. We report cross-validated balanced accuracy (BAC) to estimate generalisibility of the classifiers, which was tested against chance performance using permutation tests with 1000 label permutations. We further compared FC against EC classifiers using McNemar’s tests. Lastly, we report mean decrease in accuracy on permuted out-of-bag samples for each connection to measure feature importance.

## Results

### The effect of LSD on functional connectivity

**Fig. 1:**
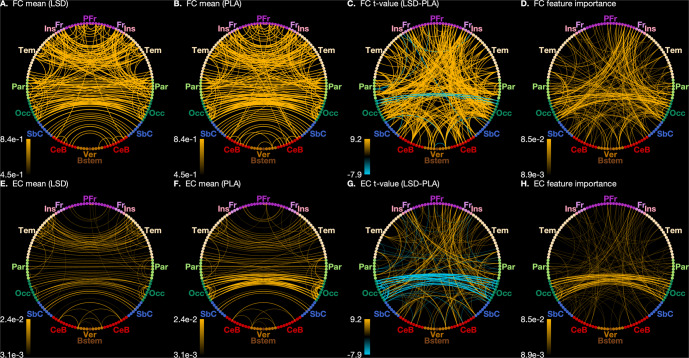
Connectogram views of differences in functional (FC) and effective connectivity (EC) between LSD and placebo conditions. **A** Across-participant average FC in the LSD condition. **B** Across-participant average FC in the placebo condition. **C** Across-participant *t*-statistic values of difference between FC in LSD and placebo conditions. **D** Feature importance estimates for the FC classification model. See ’Statistical analysis’ for a detailed definition of feature importance. **E** Across-participant average EC in the LSD condition. **F** Across-participant average EC in the placebo condition. **G** Across-participant *t*-statistic values of difference between EC in LSD and placebo conditions. **H** Feature importance estimates for the EC classification model. Differences in magnitudes of connectivity are indicated in each connectogram by both line width and opacity. In (**C**) (FC) and (**G**) (EC), orange and blue lines indicate stronger and weaker connectivity, respectively, in connectivity in the LSD condition. Note that for (**E**)–(**H**), both directional EC values between each pair of regions have been averaged for display. To maintain visibility, only the top 250 connections have been displayed. PFr Prefrontal cortex. Fr Frontal cortex. Ins Insular cortex. Tem Temporal cortex. Par Parietal cortex. Occ Occipital cortex. SbC Subcortical regions. CeB Cerebellum. Ver Vermis. Bstem Brainstem.

Mass-univariate tests suggested that about 23% (1993/8646) unique correlation coefficients significantly differed across LSD and placebo conditions (*p* < 0.05). Among these connections, we observed mostly stronger FC under LSD (Fig. 1A–C, Fig. S2).

#### Partial least squares correlation analysis

**Fig. 2:**
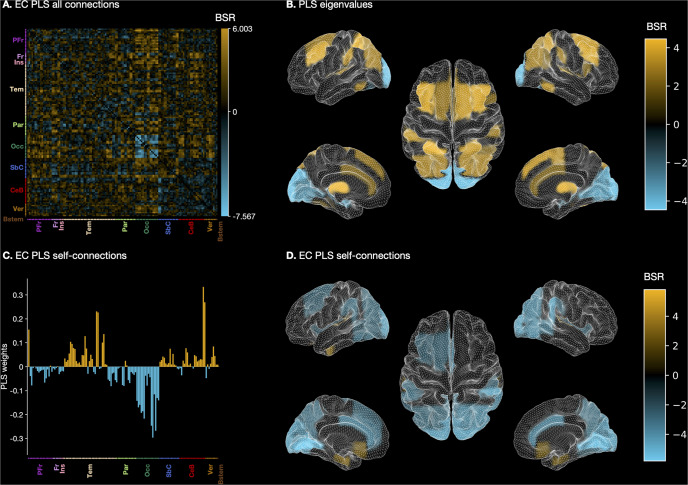
Graphical and anatomical visualisations of partial least squares (PLS) correlation analysis results. **A** Bootstrap ratios (BSRs) of whole-brain EC reflecting condition differences. BSRs are the ratios of the loadings on the latent variable and the standard errors estimated from bootstrapping. The larger the magnitude of a BSR, the larger the weight (i.e., the loading on the latent variable) and the smaller the standard error (i.e., higher stability; [55, 56]). BSRs can be understood analogous to z-scores if bootstrap distributions are approximately normal [57]. **B** Leading Eigenvector reflecting condition differences in whole-brain EC across brain regions. **C** Brain region saliences reflecting condition differences across self-connections. **D** Brain region BSRs reflecting condition differences across self-connections. For (**A**)**-**(**D**): Orange and blue areas indicate stronger and weaker connectivity respectively, respectively, under LSD compared to placebo. PFr: Prefrontal cortex. Fr: Frontal cortex. Ins: Insular cortex. Tem: Temporal cortex. Par: Parietal cortex. Occ: Occipital cortex. SbC: Subcortical regions. CeB: Cerebellum. Ver: Vermis. Bstem: Brainstem.

PLSC analysis of FC showed a significant condition effect on the first LV (LSD condition score: 4.442 [3.785, 5.076], placebo: −4.442[−5.076, −3.785], *p* < 0.001), but not on the second LV (*p* = 1.000). LV loadings indicated that FC was stronger under LSD compared to placebo across a large number of regions. The most reliable effects were observed for the following regions connections: bilateral lingual gyrus and bilateral inferior frontal gurys (pars opercularis), right inferior frontal gyrus and right lingual gyrus, left cuneus and right middle frontal gyrus (MFG), and left temporo-occipital middle temporal gyrus (MTG) and left intracalcerine cortex (bootstrap ratios (BSR) > 6.3). Conversely, weaker FC under LSD was found between several occipital regions, including left fusiform gyrus and right inferior lateral occipital cortex (LOC), supracalcarine cortex, occipital pole, and between bilateral putamen and cerebellum (BSR ≤ −5.0; Fig. 2). Behavioural PLSC revealed a significant correlation between FC measures and subjective effects (*r* = 0.775 [0.069, 0.858], *p* = 0.031, Fig. S4B). Most reliable, positive loadings were observed for several cortical regions including inferior frontal gyrus (pars opercularis) and postcentral gyrus (Fig. S4A). Conversely, most reliable negative loadings were observed for connections between occipital and cerebellar regions (Fig. S4A).

#### Machine learning analysis

The random forest trained on FC as features (FC model) discriminated between LSD and placebo with a BAC of 86% (*p* < 0.001; Table S3, Figure S3). Feature importance measures suggested that connections involving at least one occipital region (38 of the top 50 regions ranked by feature importance or at least one prefrontal region (14/50) were most relevant for this classification performance. Of the twelve connections that did not involve occipital regions, four connected frontal and parietal regions, and the remaining temporal and parietal regions (Fig. S2C). The connections remaining in the top 10 involved connections between occipital brain regions as well as connections from occipital regions to prefrontal and temporal regions (Fig. S2D).

### The effect of LSD on effective connectivity

**Fig. 3:**
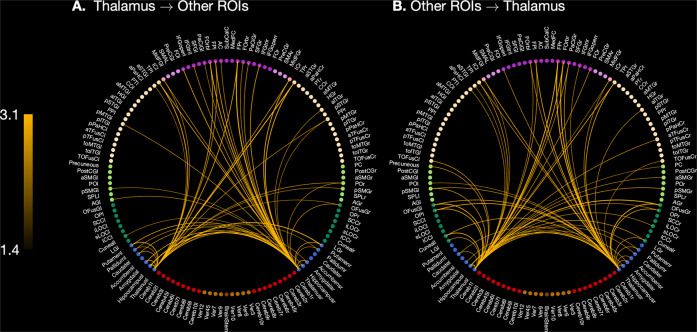
Connectogram views of thalamic effective connectivity (EC). Across-participant t-statistic values of the difference between LSD and placebo conditions in outgoing (**A**) or incoming (**B**) thalamic connections (thresholded at *p* < 0.05, whole-brain FDR-corrected). Differences in magnitudes of connectivity are indicated by both line width and opacity. Orange lines indicate stronger connectivity under LSD. Please, see Supplement for region abbreviation key.

Mass-univariate tests suggested that about 13% (2184/17424) effective connections coefficients significantly differed across conditions (*p* < 0.05). As with LSD-induced changes in FC, we observed mostly stronger EC under LSD (Fig. 1E–G, Fig. S2). However, surprisingly, among the most prominent differences was weaker EC between occipital brain regions under LSD. We further investigated how LSD impacted thalamic connectivity specifically. We found that connectivity from thalamus to other brain areas, but also from other cortical regions to thalamus was stronger (Fig. 3).

#### Partial least squares correlation analysis

PLSC analysis of EC showed a statistically significant condition effect on the first LV (LSD condition score: 0.060 [0.067, 0.052], placebo: −0.060 [−0.053, −0.067], *p* < 0.001), but not on the second LV (*p* = 1.000). Similar to the PLSC results based on FC data, we found that EC was primarily stronger under LSD. Reliable loadings were observed for connections between occipital and prefrontal regions, including inferior frontal gyrus (pars opercularis) and bilateral lingual gyrus, anterior cingulate cortex and right occipital pole, anterior cingulate cortex and right fusiform gyrus, left cuneus and right MFG, left intracalcarine cortex and left MTG, right lingual gyrus, and superior frontal gyrus, anterior cingulate cortex and right intracalcalcarine cortex, and right precentral gryus and left superior LOC (BSR > 5.5). The reverse pattern showing weaker EC under LSD was observed exclusively between several occipital regions, including the occipital pole, lingual gyrus, supracalacarine cortex, intracalcarine cortex, and fusiform gyrus (BSR < − 6.0; Fig. 2). Behavioural PLSC revealed a significant correlation between FC measures and subjective effects (*r* = 0.932[0.906, 0.957], *p* = 0.047, Fig. S4E). Most reliable, positive loadings were observed for connections between several cortical areas, such as angular gyrus and inferior frontal gyrus (pars triangularis) as well as amygdala (Fig. S4D). Conversely, most reliable negative loadings were observed for connections between occipital and cerebellar regions (Fig. S4D).

#### Machine learning analysis

The random forest trained on EC as features (EC model) performed with a BAC of 91.1% (*p* < 0.001; Table S3, Fig. S3). As with the mass-univariate and PLSC analysis, connections involving occipital regions were the most predominant features driving the classification performance (Fig. S2G, H). Of the top 50 connections (ranked by feature importance), the majority (39/50) represented connections between pairs of occipital regions. The highest-ranked connection involving a non-occipital region included right dorsolateral PFC to right lingual gyrus and left dorsolateral PFC to left cerebellum. This finding suggests that these connections were not only significantly different under LSD (Fig. 1G), but also contributed substantially to highly-accurate individual-level discrimination performance.

### Comparing functional vs effective connectivity changes under LSD

#### The effect of LSD on inhibitory self-connections

As pointed out in the Introduction, an advantage of using EC over FC is that rDCM allows estimation of inhibitory self-connections (see Supplement). These all-negative values can be interpreted as ‘decay-rate’ coefficients (in view of the DCM state equation) since they capture the tendency of different regions to return to baseline, rather than increasing activity indefinitely. Also note that these values reflect local as opposed to global (inter-regional) dynamics.

**Fig. 4:**
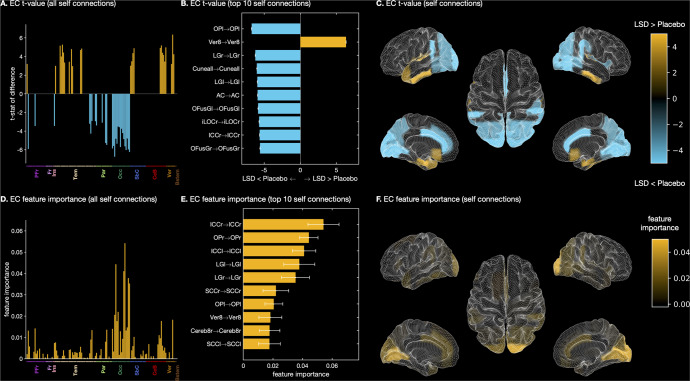
Effect of LSD on inhibitory self-connections. **A** t-statistic of the difference between LSD and placebo conditions in self-connections. **B** Top 10 self-connections ranked by t-statistic of the difference between LSD and placebo conditions. **C** Anatomical colourmap of t-statistic of the difference between LSD and placebo conditions in self-connections. **D** Estimates of feature importance of self-connections in EC classification model. **E** Top 10 self-connections by feature importance in the EC classification model. **F** Anatomical colourmap displaying feature importance of self-connections in the EC classification model. For (**A**), (**C**): Orange and blue areas indicate stronger and weaker connectivity under LSD, respectively. For (**B**), errorbars represent the across-participant standard deviation of the differences in connectivity between conditions. In (**B**) and (**E**), abbreviations indicate the ROIs forming each connection. For (**E**), errorbars represent the across-fold standard deviation of the feature importance estimates. PFr Prefrontal cortex, Fr Frontal cortex, Ins Insular cortex, Tem Temporal cortex, Par Parietal cortex, Occ Occipital cortex, SbC Subcortical regions, CeB Cerebellum, Ver Vermis, Bstem Brainstem.

Mass-univariate tests suggested that about 30% (39/132) inhibitory self-connections significantly differed across conditions (*p* < 0.05). Occipital regions once again displayed the greatest effects showing more negative local connectivity under LSD (Fig. 4, Fig. S2).

*Partial least squares correlation analysis*. LSC analysis performed on self-con-nections showed a significant condition effect on the first LV (LSD condition score: 0.0004 [0.00003, 0.00005], placebo: −0.0004 [−0.0005, −0.0003], *p* < 0.001), but not on the second LV (*p* = 1.000). Self-connections were more inhibitory (i.e., became more negative) under LSD primarily in occipital, parietal, and prefrontal regions (Fig. 2C4). Stronger self-inhibition under LSD occurred in the bilateral lingual gyri, intracalcarine cortex, fusiform gyri, occipital pole, cuneus, posterior supramarginal gyrus and supracalcarine cortex (Fig. 2D). Conversely, disinhibition under LSD was observed in temporal, subcortical and brainstem regions (Fig. 2D). The largest disinhibition under LSD was noted in the cerebellum, bilateral pallidum, parahippocampal gyri, and inferior temporal gyrus (Fig. 2D).

Note that all self-connections are negative. Disinhibition in any individual brain area suggests that this area becomes less stable under LSD from a dynamic system viewpoint, since the system moves closer to a critical point as self-connections approach zero.

*Machine learning analysis* Restricting the feature rankings of the EC model (see ‘Machine learning analysis’) to self-connections, we found again that many of the most important connections were occipital. Among the top 10 self-connections ranked by feature importance were two non-occipital connections, namely Vermis 8 and right Cerebellum 8 (Fig. 4).

#### Asymmetry in directed connectivity

**Fig. 5:**
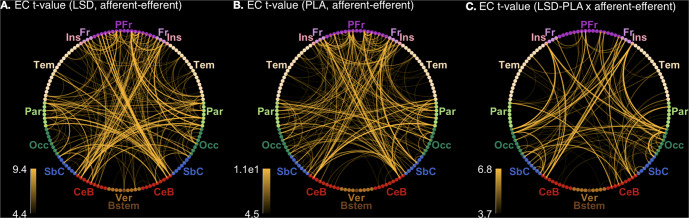
Connectogram views of asymmetries in effective connectivity (EC). **A** Across-participant t-statistic of the difference in EC between the two directions of influence between each pair of regions, for the LSD condition. **B** Across-participant t-statistic of the difference in EC between the two directions of influence between each pair of regions, for the placebo condition. **C** Across-participant t-statistic of the difference in EC between the two directions of influence between each pair of regions, and between the LSD and placebo conditions. Differences in magnitudes of connectivity and connectivity changes are indicated in each connectogram by both line width and opacity. To maintain visibility, only the top 250 connections have been displayed. PFr Prefrontal cortex, Fr Frontal cortex, Ins Insular cortex, Tem Temporal cortex, Par Parietal cortex, Occ Occipital cortex, SbC Subcortical regions, CeB Cerebellum, Ver Vermis, Bstem Brainstem.

Recall that while FC provides a measure of the correlation between the activities of each pair of brain regions, EC provides an estimate of directed effects between pairs of regions, which may be asymmetrical. We tested for the presence of asymmetry (by mass-univariate comparison) in each condition (LSD and placebo), and tested whether asymmetry was affected by LSD (drug-by-asymmetry interaction). The number of forward-backward pairs of endogenous connectivity coefficients that were significantly different (*p* < 0.05) was 868 out of 8648 (10%) under LSD, 1202 (14%) in the placebo condition, and 120 (1.4%) for the drug-by-asymmetry interaction (Fig. 5).

#### Comparing functional and effective connectivity classifiers

Lastly, we compared the performances of classifiers trained on either FC or EC to test whether modelling additional physiological details (i.e., self-inhibition and asymmetry) translated into a better classification performance of drug condition. While BACs differed numerically between the FC and EC classification models (86% vs 91% BAC), a McNemar’s test comparing the models’ performances suggested that these differences were not significantly different (*p* > 0.074), indicating that both FC and EC may be promising biomarkers for individual-level predictions.

## Discussion

The goal of this study was to investigate the effects of LSD on whole-brain EC and gauge its potential as a biomarker to decode or predict subjective effects in the future. To this end, we studied LSD-induced effects on whole-brain EC through multivariate, and machine-learning analyses and compared EC to FC. Multivariate PLSC analyses revealed stronger EC between parietal, temporal and inferior frontal regions under LSD, but weaker EC between regions in occipital cortices. When comparing FC to EC, we found notable changes in self-inhibition in around 30% of the brain regions under LSD, indicating that LSD may perturb the excitation/inhibition (E/I) balance of the brain. Moreover, our results suggest that—while EC was asymmetric (10–14% of connections)—asymmetry was largely unaffected by LSD (1.4% of connections). Lastly, our behavioural PLS and machine learning analyses showed that both FC and EC constitute promising biomarkers for future individual-level predictions of subjective effects. These analyses revealed that changes in FC and EC between several regions including angular gyrus and inferior frontal gyrus as well as connections between occipital and cerebellar regions correlated with global subjective effects of LSD and classifiers trained on either FC or EC could discriminate between LSD and placebo with high accuracy of 86% and 91%, respectively.

### Face validity of whole-brain effective connectivity

To assess the face validity of EC measures, we compared thalamic connections and found that the effects (sign and significance) were consistent across FC and EC, although a recent study argued that this does not necessarily need to be the case [16]. Indeed, changes in thalamic connectivity across conditions were also in perfect agreement with those of a previous FC analysis based on a subset of participants [14]. Together with the highly accurate classification performance, these findings reassured us that EC estimates appeared to show face validity, i.e. EC estimates were consistent with previous results and affected by LSD.

Comparing all connections, we found that FC and EC measures were broadly in agreement with one another as both indicated generally stronger connectivity under LSD, with some notably weaker connectivity between bilateral occipital areas, though this effect was more pronounced in the EC measure. Feature importance also suggested that the EC classification relied more heavily on the bilateral occipital connections than the FC classification, wherein connections involving more varied areas were represented among the highlighted ‘important` features (Figure S2).

### Comparison with other studies investigating directed connectivity

Unlike another study that investigated directed connectivity using Granger causality based on magnetoencephalography (MEG) recordings [16], we found predominantly stronger EC mirroring stronger FC patterns across the brain with the exception of the aforementioned occipital connections. This difference may be explained by the different methods used (Granger causality measuring directed FC vs rDCM measuring EC) or the measurement modalities and resulting differences in temporal resolution (MEG vs fMRI).

Moreover, [20] investigated EC in cortico-striato-thalamo-cortical feedback loops in a sparse network to test the thalamic gating hypothesis using spectral DCM. Their chosen network consisted of the thalamus, ventral striatum (VS), posterior cingulate cortex (pCC), and temporal cortex. The authors reported stronger 5-HT_2A_-receptor-dependent thalamus → pCC connectivity and weaker 5-HT_2A_-receptor-independent VS → thalamus connectivity under LSD. In line with their results, we also found stronger thalamus → pCC connectivity under LSD, while weaker VS → thalamus connectivity was not reproduced in our study. In our data, the right accumbens → right thalamus connection was stronger under LSD. However, weaker VS → thalamus was independent of 5-HT_2A_-receptor antagonist Ketanserin administration in [20], while stronger thalamus → pCC connectivity was observed when the 5-HT_2A_ was not blocked—perhaps pointing to differing mechanisms of action.

### Implications for the thalamic gating hypothesis and brain entropy accounts

Our results are in line with the thalamic gating hypothesis, which postulates that psychedelics may temporally reduce thalamic gating leading to excessive information flow from thalamus to cortical regions [36]. As previous analyses investigating FC [10, 14] and EC [20] changes under LSD, we found stronger connectivity from thalamus to a widespread network of cortical regions Fig. 3. Interestingly, connectivity from cortex to thalamus was also stronger under LSD, which possibly suggests the presence of positive feedback loops between thalamus and cortex.

Brain entropy accounts of psychedelics propose that altered states of consciousness observed following administration of psychedelics result from increased entropy in the brain [37–39]. Even though testing these accounts was not the goal of this study, we note that overall our results are in agreement with this proposal. Specifically, we found changes in self-connections or within-region dynamics suggesting widespread disinhibition across most of the cortex. From a dynamic system perspective, this disinhibtion renders the system more unstable since it approaches a critical point as self-connection values approach zero. However, it is worth noting that we found the opposite pattern in occipital regions, where local inhibition increased under LSD rendering dynamics in these areas more stable. While our results are generally in line with the proposal that psychedelics like LSD increase brain entropy (on average), this whole-brain analysis revealed that a more fine-grained assessment of entropy across different regions may be warranted in future studies to refine these accounts.

It is interesting to note that connections between occipital regions appear to dominate EC under placebo (Fig. 1F), whereas the difference between visual and other regions is reduced under LSD (Fig. 1E). This result aligns with a recent study [40], which showed that the principal gradient of cortical connectivity flattens under LSD. Our results support the authors’ interpretation of a decrease in functional differentiation between sensory and abstract cognitive processing under LSD.

### Does LSD perturb the excitation/inhibition (E/I) balance?

Recent studies have begun to investigate the impact of psychedelics on glutamate-mediated excitation of the cortex. At least two different pathways for glutamatergic effects have been proposed: (1) Agonism at 5-HT_2A_ receptors on pyramidal cells may lead to increased glutamate release [41, 42] and (2) agonism at postsynaptic, inhibitory 5-HT_1A_ receptors may result in reduced excitation in certain regions, for example in hippocampus [43, 44]. While we cannot directly speak to these pathways, widespread increases in self-connections or local disinhibition as identified in this study may relate to 5-HT_2A_-mediated glutamate release. Conversely, increased inhibition could be mediated via the 5-HT_1A_ pathway, although this receptor is not strongly expressed in occipital regions [45]. Rather, 5-HT_1B_ expression is increased in these regions [45] and may be a candidate mechanism requiring further investigation.

Our results along with preclinical studies [42, 46, 47] and recent magnetic resonance spectroscopy results [44] suggest that LSD may perturb the E/I balance of the brain. This is especially relevant because disturbances in the E/I balance have been discussed in the context of psychosis [48, 49] and more recently in the context of psychedelic-induced hallucinations and synaesthesia [50]. Comparing LSD-induced changes in E/I balance to other psychedelics and those associated with clinical psychosis will be an interesting avenue for future research.

### Limitations

A few limitations of this study merit attention. Despite a washout period of at least two weeks, we cannot fully exclude carry-over effects due to potential long-term effects of psychedelics [51]. An additional limitation in this context is the variable time between placebo and LSD sessions, which was owed to constraints in scheduling participants, since the study required participants to spend 24h under supervision on each study day. We also cannot exclude that participants fell asleep during placebo scans. Further limitations are the single post-drug scan design and the small sample size.

Moreover, LSD is known to affect heart rate, blood pressure and body temperature [52, 53]. Here, we controlled for physiological effects using baseline measures. Nonetheless, these effects are likely still impacting connectivity estimates and may partially account for the effects. Future studies should include concurrent physiological measurements during MRI acquisition to allow for more detailed physiological noise modelling (e.g., see [54]).

Due to the salient subjective effects of LSD, blinding is inherently difficult. Thus, knowledge about the condition could have impacted neural effects and future studies should include active control conditions. Although do note that one of the studies included an active control [30], rendering it less likely that participants correctly identified the condition in part of the data. A limitation of the behavioural PLSC analysis is that subjective effects were rated retrospectively and not restricted to the time window of the fMRI measurements.

Finally, while classification performances in this study were promising, they should be taken as preliminary until replicated in an external sample.

### Future directions

Our results suggest that local gain is changed under LSD implicating disturbances of the E/I balance as a neural mechanism underlying LSD effects. Because rDCM summarises region-specific E/I balance by a single parameter per region, we cannot determine whether excitation, inhibition, or both are impacted. Future studies should employ more detailed models that allow to pinpoint these changes and compare them empirically to other conditions in which the E/I balance is affected, for example psychosis [49, 50].

Furthermore, our results suggest that visual regions may be impacted quite differently both in terms of between-region connectivity as well as in terms of local gain compared to the rest of the cortex warranting further investigation. The physiological basis of these changes and the relationship with subjective effects—visual hallucinations in particular—should be examined in future studies. A comparison of eyes-open vs eyes-closed resting states would be valuable to determine whether the EC changes observed here are a reflection of eyes-open-like behaviour of the visual system.

Finally, we found that both whole-brain FC and EC were equally capable of discriminating between LSD and placebo with high accuracy suggesting that both are promising candidates for more challenging prediction targets. More research is needed to assess whether machine learning models trained on either FC and EC estimates are able to predict subjective effects of LSD at an individual level or from baseline EC measured before LSD intake.

### Conclusions

To the best of our knowledge, this is the first study to examine the impact of LSD on whole-brain EC. We found that compared to placebo, LSD impacted local gain and was associated with primarily stronger FC and EC with the notable exception of connections involving occipital and subcortical regions. Moreover, EC correlated with global subjective effects and discriminated experimental conditions with high accuracy (91.11%) highlighting that EC preserved classification accuracy while providing additional mechanistic information pointing towards LSD-induced disturbances of the E/I balance. This result suggests that EC is a promising candidate biomarker to decode or predict subjective effects of LSD in the future.

## Supplementary information

Supplementary Material
