# Supplementary material for: The effect of lysergic acid diethylamide (LSD) on whole-brain functional and effective connectivity
Source: Neuropsychopharmacology. 2023 Apr 25;48(8):1175–83. doi: 10.1038/s41386-023-01574-8 (PMC10267115; doi:10.1038/s41386-023-01574-8)
Supplement: Supplementary file 1 — Supplementary Material [file 41386_2023_1574_MOESM1_ESM.pdf]

Supplementary Material: The effect of lysergic acid diethylamide (LSD) on whole-brain functional and effective connectivity

Peter Bedford[1*], Daniel J. Hauke[2*], Zheng Wang[1], Volker Roth[3], Monika Nagy-Huber[3], Friederike Holze[4], Laura Ley[4], Patrick Vizeli[4], Matthias E. Liechti[4], Stefan Borgwardt[5], Felix Müller[6+], Andreea O. Diaconescu[1,7,8,9+]

1 Krembil Centre for Neuroinformatics, Centre for Addiction and Mental Health (CAMH), Toronto, Ontario, Canada
2 Centre for Medical Image Computing, Department of Computer Science, University College London, London, United Kingdom
3 Department of Mathematics and Computer Science, University of Basel, Basel, Switzerland
4 Division of Clinical Pharmacology and Toxicology, Department of Biomedicine and Department of Clinical Research, University Hospital Basel, University of Basel, Basel, Switzerland
5 Department of Psychiatry and Psychotherapy, Translational Psychiatry, Lübeck, Germany
6 Department of Psychiatry (UPK), University of Basel, Basel, Switzerland
7 Department of Psychiatry, University of Toronto, Toronto, Ontario, Canada
8 Institute of Medical Sciences, University of Toronto, Toronto, Ontario, Canada
9 Department of Psychology, University of Toronto, Toronto, Ontario, Canada
***** Peter Bedford and Daniel J. Hauke are joint first authors.
+ Felix Müller and Andreea O. Diaconescu are joint last authors.

Contents

1 Supplementary Methods **3**
1.1 Participants . . . 3
1.1.1 Sample size and recruitment . . . 3
1.1.2 Inclusion and Exclusion criteria . . . 3
1.2 Data acquisition . . . 4
1.3 Preprocessing . . . 4
1.4 Effective Connectivity Estimation . . . 5
1.5 Covariate correction . . . 6
1.6 Machine learning analysis . . . 6
1.7 Software resource table . . . 7
1.8 Baseline physiology measures . . . 8
1.9 Region of interest label abbreviation key . . . 9

2 Supplementary Results **10**
2.1 Assessment of feature collinearity . . . 10
2.2 Feature rankings by t-test and feature importance . . . 10
2.2.1 The effect of LSD on functional connectivity . . . 10
2.2.2 The effect of LSD on effective connectivity . . . 12
2.2.3 The effect of LSD on inhibitory self-connections . . . 12
2.3 Classification performances . . . 12
2.4 Behavioral PLSC results . . . 13

1 Supplementary Methods

1.1 Participants

Data was aggregated across two randomized, placebo-controlled, double-blind, cross-over trials. We will briefly reiterate the most important aspects pertaining to our analysis below. Please, refer to the original publications for further details (trial A: [1, 2]; trial B: [3]). Both studies were approved by the Ethics Committee for Northwest/Central Switzerland (EKNZ) and by the Federal Office of Public Health. All participants provided written consent prior to participating and received monetary compensation.

1.1.1 Sample size and recruitment

Participants were recruited from the University of Basel or by advertisement and word of mouth. In trial A, 20 healthy participants (10 male, 10 female; age 32 ± 11 [mean $\pm$ SD]; range $25 - 60$ years; body weight, $68.8 \pm 7.7kg$) were selected out of initially 23 participants for which functional magnetic resonance imaging (fMRI) data was recorded ($N = 3$ participants were excluded from analysis due to excessive head motion; see Preprocessing Section). Only two participants had used a hallucinogenic drug before, and both on only a single occasion. In trial B, 25 out of initially 28 healthy participants (12 men, 13 women; age 28 ± 4.3; range $25 - 45$ years; body weight, $70.2 \pm 11.2kg$) were analysed ($N = 1$ participant was excluded because unblinding revealed that they accidentally did not receive LSD and $N = 2$ participants were excluded from analysis due to excessive head motion; see Preprocessing Section). Five participants had previously used a hallucinogen (5-HT_{2A} agonists), including LSD (three participants, $1 - 4$ times). Eight participants had never used any illicit drugs with the exception of cannabis. The final sample comprised 45 participants collected from Trials A and B.

1.1.2 Inclusion and Exclusion criteria

Participants between the ages of $21 - 65$ years (trial A) and $25 - 50$ years (trial B) were recruited to take part in the study. Exclusion criteria for both studies were: Age < 25, pregnancy, personal or first-degree relative history of major psychiatric disorders (assessed by the Semi-structured Clinical Interview for Diagnostic and Statistical Manual of Mental Disorders, 4th edition, Axis I disorders by a trained psychiatrist), tobacco smoking > 10 cigarettes/day, use of medications that may interfere with study medications (e.g. antidepressants, antipsychotics, sedatives), lifetime prevalence of illicit drug use > 10 times (excluding Δ^9- tetrahydrocannabinol), illicit drug use within the last two months, and illicit drug use during the study (determined by urine drug tests).

Study-specific exclusion criteria included: Age > 65, history of drug dependence and nursing, cardiac or neurological disorders, hypertension ($> 140/90mmHg$) or hypotension (SBP$< 85mmHg$) in trial A and age > 50, chronic or acute physical illness (abnormal physical exam, electrocardiogram, or hematological and chemical blood analyses) in trial B. Both studies included general health assessments.

1.2 Data acquisition

All images were acquired using a 3 Tesla MRI system (Magnetom Prisma, Siemens Healthcare, Erlangen, Germany) with a 20-channel phased array radio frequency head coil. For anatomical images a T1-weighted MPRAGE sequence (field-of-view: 256×256mm^2; resolution: $1 \times 1 \times 1$mm^3; TR: $2000ms$; TE: $3.37ms$; flip angle: 8°; bandwidth: $200Hz$/pixel) was used. Resting-state fMRI was acquired using interleaved T2*-weighted EPI using the following parameters: 35 axial slices, slice thickness: 3.5mm, inter-slice gap: 0.5mm, field-of-view: 224×224cm^2, resolution: $3.5 \times 3.5 \times 3.5mm^3$, TR: $1800ms$, TE: $28ms$, flip angle: 82°, and bandwidth: $2442Hz$/pixel. Three hundred volumes were acquired (total acquisition time: $9min$). This resting state scan was followed by other sequences not relevant for this publication. Total acquisition time for all sequences was approximately 50min. No significant differences in head motion were present between conditions (see [2] for more details).

1.3 Preprocessing

Preprocessing settings were kept identical to previous analyses [2, 4] to ensure comparability and enable us to assess the face validity of this new modelling approach by comparing it to previous results. Structural and functional neuroimaging data were preprocessed using the CONN toolbox [5] based on SPM12 [6] (see all software resources in Table S1). The first five volumes of the BOLD time series were discarded to ensure signal equilibrium. Temporal and spatial preprocessing included slice-timing correction, realignment to the mean image, and co-registration to the participant's own structural scan. The structural image underwent a unified segmentation procedure combining segmentation, bias correction, and spatial normalization [7]. The same normalization parameters were then applied to the functional images. Functional images were smoothed with an isotropic Gaussian kernel (6mm FWHM).

Quality control assessments comprised three stages: First, all scans were assessed considering the percentage of scrubbed volumes. Scrubbing was performed at a global signal threshold of $z > 3$ and a composite subject motion threshold of > 0.5mm. Participants were excluded if < 5min of the scan remained after scrubbing (corresponding to $< 55\%$ of the initial volumes). This was due to evidence indicating that resting-state scans < 5min are not reliable [8]. No participant was excluded based on this criterion. Second, maximal head motion after the scrubbing procedure were assessed using frame-wise displacement (FD) [9]. A sphere radius of 50mm was chosen for the calculation, and participants were excluded if maximum FD was > 1.75mm (half-voxel size). Five participants were excluded based on this criterion, resulting in a final sample of 45 participants. The mean percentage of the scrubbed volumes for this sample was $5.5 \pm 3.7\%$ for the drug condition and $3.8 \pm 2.6\%$ for the placebo condition. Average maximum FD was $0.74 \pm 0.39mm$ for the drug condition and $0.49 \pm 0.24mm$ for the placebo condition.

Further noise correction of the functional images included linear regression of the six motion parameters and the white matter and cerebrospinal fluid signals, using individual tissue masks obtained from the T1-weighted structural images. The resulting

functional images were band-pass filtered ($0.008\text{Hz} < f < 0.09\text{Hz}$). Regional time-series were extracted based on a parcellation according to the Harvard-Oxford cortical and subcortical structural atlas (48 cortical regions: 43 bilateral, 5 others; 25 subcortical regions: 16 bilateral, 9 others; totaling $132 = 43 \times 2 + 5 + 16 \times 2 + 9$). This parcellation was chosen based on previous recommendations [10] to ensure comparability with other analyses [4] and evaluate the face validity of the rDCM approach.

1.4 Effective Connectivity Estimation

Regression dynamic causal modelling (rDCM) is a reformulation of the original DCM for fMRI framework [11]. In general, DCM describes the brain as a non-linear dynamic system, which can be summarized by two equations: a neural state equation and a (non-linear) observer equation. In classic DCM, the state equation describes how activity in brain regions evolves over time, as a function of endogenous connectivity between brain regions, changes in connectivity and activity induced by experimental inputs. In this study, we modelled task-free data and did not consider external experimental inputs. The observer equation describes how changes in neuronal states translate into changes in measurements. For fMRI data, this is a non-linear hemodynamic model [12–15]. Under suitable assumptions and choice of prior distributions over the parameters, the likelihood function can be specified and the model inverted to estimate effective connectivity from fMRI data.

In classic DCM, analyses need to be restricted to model networks of no more than a few regions using experiments, which carefully control experimental perturbations of network activity to ensure model invertibility [16]. To meet these practical constraints *a priori* knowledge of the system under analysis is required. In contrast, rDCM enables inference of EC in whole-brain networks. Through a series of simplifications including, crucially, the transformation into the frequency- (rather than time-) domain, the original DCM model can be recast as a linear regression in the frequency-domain (hence regression or rDCM) reducing its concomitant computational burden and overcoming some of the limitations of classic DCM [11]:

$$\mathbf{Y} = \mathbf{X}\boldsymbol{\theta} + \boldsymbol{\nu}, \tag{1}$$

with

$$\boldsymbol{\nu} \sim \mathcal{N}\left(\boldsymbol{\nu}; 0, \boldsymbol{\tau}^{-1}\mathbf{I}_{N\times N}\right), \tag{2}$$

where $\mathbf{Y}$ is the independent variable (proportional to the Fourier-transformed BOLD signals $\widehat{\mathbf{y}}_i$); $\mathbf{X}$ represents the design matrix, which consists of the regressors (the Fourier-transformed BOLD signals); $\boldsymbol{\theta}$ is the parameter vector, which consists of the endogenous connectivity strengths a_{ij} from region i to region j, and $\boldsymbol{\nu}$ is the noise, with precision τ_i^{-1} distinct for each region. Note, that for $i = j$, a_{ij} corresponds to a connection from region to itself (i.e., a self connection), which are assumed to be inhibitory to render the dynamic system stable. We refer to these connectivity coefficients when speaking about inhibitory self-connections.

Additional simplifications from the original DCM framework include: (i) replacing the nonlinear biophysical model of hemodynamics with a linear hemodynamic response function, (iii) applying a mean field approximation across regions (i.e., connectivity parameters targeting different regions are assumed to be independent), and (iv) specifying conjugate priors on neuronal (i.e., connectivity and driving input) parameters and noise precision. The face- and construct-validity of rDCM for use with rs-fMRI data was recently demonstrated by [17].

1.5 Covariate correction

Covariate correction for both partial least square correlation analysis (PLSC) and the random forest analysis entailed first explaining variance in the connectivity features of interest (for example, effective connectivity) using a linear model for each feature f:

$$\begin{aligned} f_{uncorr} &= \beta_0 + \beta_1 \times \text{heart rate} + \beta_2 \times \text{diastolic blood pressure} \\ &+\beta_1 \times \text{systolic blood pressure} + \beta_4 \times \text{body temperature} + R, \end{aligned} \tag{3}$$

where R is the residual that cannot be explained by any of the covariates. We then replace the original features with these residual values:

$$f_{corr} := R \tag{4}$$

These corrected features only contain the variance that cannot be explained by a linear combination of the covariates. Baseline measures were taken prior to drug administration approximately three hours before the resting state sequence (150.4 ± 13.0 min, [mean ± SD]). Note, that we did not regress out physiological measures following drug administration, because these measures cannot be considered covariates, since they are affected by the intervention [18] (usually this implies that the variable can be measured before the intervention); rather physiological measurements following drug administration should be considered effects of the intervention. We discuss this limitation of our work in the main manuscript.

1.6 Machine learning analysis

To assess how well condition (LSD versus placebo) could be classified from whole-brain connectivity signatures, we trained two random forest classifiers [19] on either whole-brain FC or EC as features to classify conditions. Random forests were chosen, because they work well out-of-the-box and did not require further hyperparameter tuning, which would have entailed a further split of the already small sample for the purpose of hyperparameter optimization. The number of decision trees was kept constant and equivalent to the number of features for each model without further optimization (due to the computational constraints and the high-dimensionality of the data). All other parameters were kept to the default values of the function. Both classifiers were implemented using the 'TreeBagger' function.

Preprocessing of connectivity features consisted of (i) pruning features with zero variance and (ii) covariate correction for baseline heart rate, diastolic blood pressure, systolic blood pressure and body temperature (see for example, [20]). Preprocessing was embedded in a 5-fold cross-validation to estimate the generalisability of the classification models, i.e., regression coefficients for each covariate were only fitted in the training data and then applied to the test data without refitting regression models, to prevent information leakage into the test set. Since data was collected using a within-subject design, we additionally ensured that both conditions of a given person were included either in the training *or* the test set to exclude that generalisibility estimates were influenced by person-specific information and prevent potential information leakage. We report accuracy (ACC), balanced accuracy (BAC), sensitivity (SE), specificity (SP), positive and negative predictive values (PPV and NPV), receiver operating characteristic (ROC) curves, and area-under-the-curve (AUC) to evaluate performance of both classifiers (see Table SS3). To test whether classifiers performed significantly above-chance we conducted permutation tests with 1000 label permutations. Furthermore, we compared classifiers that were trained on different feature sets (FC, EC) against each other using McNemar's tests.

Lastly, to assess which features were most relevant to distinguish LSD from placebo conditions, we computed the mean decrease in accuracy on permuted out-of-bag samples for each connection. This measure indicates how much performance decreases when removing a feature from the model and reflects the importance of this feature for the overall classification performance (a larger decrease in accuracy indicates higher importance).

1.7 Software resource table

Analysis	Resource	Source	Version	URL
Preprocessing	MATLAB	The MathWorks, Inc.	2019b	`www.mathworks.com/`
Preprocessing & FC	CONN toolbox	Whitfield et al. (2012) [5]	19c	`https://web.conn-toolbox.org/`
Preprocessing	SPM12	Friston et al. (2007) [6]	7487	`https://www.fil.ion.ucl.ac.uk/spm/`
rDCM	MATLAB	The MathWorks, Inc.	2018a	`www.mathworks.com/`
rDCM	TAPAS	Frässle et al. (2021) [21]	4.40	`www.translationalneuromodeling.org/tapas`
FDR	MATLAB	Grope (2015) [22]	2.3.0.0	`www.mathworks.com/matlabcentral/fileexchange/27418-fdr_bh`
PLSC	python	Python Software Foundation [23]	3.7.15	`www.python.org/`
PLSC	pyls	pyls developers	d8a19d5	`www.github.com/rmarkello/pyls`

Table S1: **Software resource table**. rDCM: regression dynamic causal modelling [16, 17, 24]. FDR: false discovery rate [25]. PLSC: partial least square correlation analysis [26, 27].

1.8 Baseline physiology measures

heart rate			systolic blood pressure			diastolic blood pressure			temperature		
LSD mean [SD]	PLA mean [SD]	Statistic	LSD mean [SD]	PLA mean [SD]	Statistic	LSD mean [SD]	PLA mean [SD]	Statistic	LSD mean [SD]	PLA mean[SD]	Statistic
68.50 [11.90]	65.92 [10.98]	$t = 1.534$ $p = 0.132$	123.63 [10.50]	119.74 [7.71]	$t = 2.692$ **p = 0.010**	74.91 [7.69]	73.51 [7.71]	$t = 1.186$ $p = 0.242$	36.85 [0.39]	36.82 [0.40]	$t = 0.551$ $p = 0.584$

Table S2: **Baseline physiology measures**. Reported are results of one-sample paired t-tests between LSD and placebo (PLA) conditions. Bold print highlights p-values significant at: $p < 0.05$, uncorrected.

1.9 Region of interest label abbreviation key

Abbreviation	ROI
AC	Cingulate Gyrus, anterior division
Accumbens	Accumbens
AG	Angular Gyrus
aITG	Inferior Temporal Gyrus, anterior division
aMTG	Middle Temporal Gyrus, anterior division
Amygdala	Amygdala
aPaHC	Parahippocampal Gyrus, anterior division
aSMG	Supramarginal Gyrus, anterior division
aSTG	Superior Temporal Gyrus, anterior division
aTFusC	Temporal Fusiform Cortex, anterior division
Brain-Stem	Brain-Stem
Caudate	Caudate
Cereb1	Cerebellum Crus1
Cereb10	Cerebellum 10
Cereb2	Cerebellum Crus2
Cereb3	Cerebellum 3
Cereb45	Cerebellum 4 5
Cereb6	Cerebellum 6
Cereb7	Cerebellum 7b
Cereb8	Cerebellum 8
Cereb9	Cerebellum 9
CO	Central Opercular Cortex
Cuneal	Cuneal Cortex
FO	Frontal Operculum Cortex
FOrb	Frontal Orbital Cortex
FP	Frontal Pole
HG	Heschl's Gyrus
Hippocampus	Hippocampus
IC	Insular Cortex
ICC	Intracalcarine Cortex
IFG oper	Inferior Frontal Gyrus, pars opercularis
IFG tri	Inferior Frontal Gyrus, pars triangularis
iLOC	Lateral Occipital Cortex, inferior division
LG	Lingual Gyrus
MedFC	Frontal Medial Cortex
MidFG	Middle Frontal Gyrus
OFusG	Occipital Fusiform Gyrus
OP	Occipital Pole
PaCiG	Paracingulate Gyrus
Pallidum	Pallidum
PC	Cingulate Gyrus, posterior division
pITG	Inferior Temporal Gyrus, posterior division
pMTG	Middle Temporal Gyrus, posterior division
PO	Parietal Operculum Cortex
PostCG	Postcentral Gyrus
PP	Planum Polare
pPaHC	Parahippocampal Gyrus, posterior division
PreCG	Precentral Gyrus
Precuneous	Precuneous Cortex
pSMG	Supramarginal Gyrus, posterior division
pSTG	Superior Temporal Gyrus, posterior division
PT	Planum Temporale
pTFusC	Temporal Fusiform Cortex, posterior division
Putamen	Putamen
SCC	Supracalcarine Cortex
SFG	Superior Frontal Gyrus
sLOC	Lateral Occipital Cortex, superior division
SMA	Juxtapositional Lobule Cortex -formerly Supplementary Motor Cortex-
SPL	Superior Parietal Lobule
SubCalC	Subcallosal Cortex
Thalamus	Thalamus
TOFusC	Temporal Occipital Fusiform Cortex
toITG	Inferior Temporal Gyrus, temporo-occipital part
toMTG	Middle Temporal Gyrus, temporo-occipital part
TP	Temporal Pole
Ver10	Vermis 10
Ver12	Vermis 1 2
Ver3	Vermis 3
Ver45	Vermis 4 5
Ver6	Vermis 6
Ver7	Vermis 7
Ver8	Vermis 8
Ver9	Vermis 9

2 Supplementary Results

2.1 Assessment of feature collinearity

We assessed collinearity between the random forest features (see Supplementary Figure S1). Collinearity did not appear to be a major concern, as only 3.2% or 1.3% of FC and EC features, respectively, show correlations with $|r| \geq 0.30$ (at least medium effect size) and only 0.23% or 0.058% show correlations with $|r| \geq 0.50$ (large effect size).

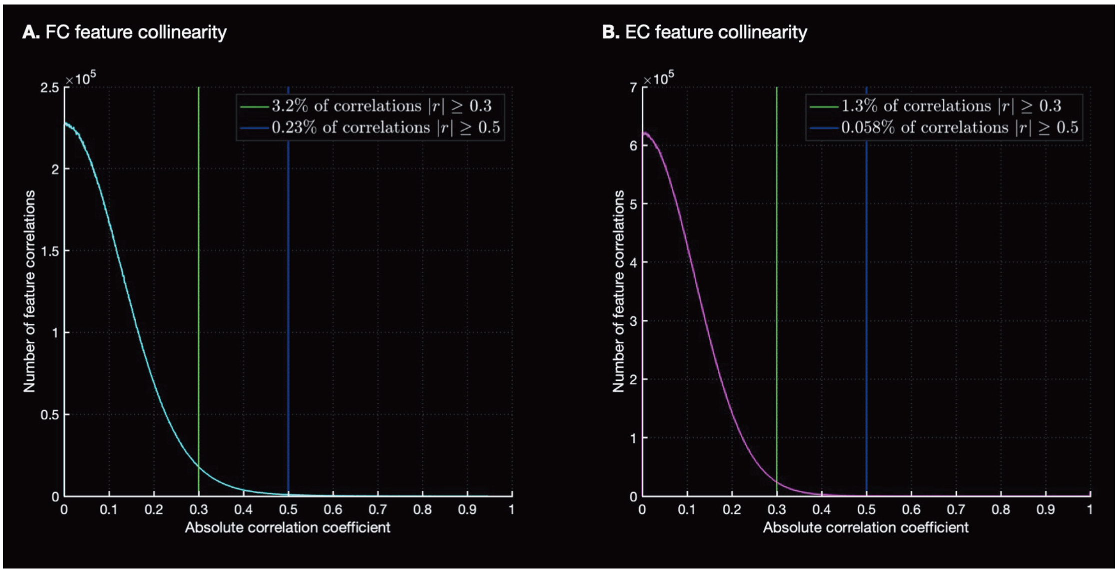

Figure S1: **Histogram of absolute correlation coefficients $|r|$ of connectivity features**. **A** Feature collinearity of functional connectivity (FC). **B** Feature collinearity of effective connectivity (EC). The histogram counts the absolute values of the correlation coefficients between every pair of effective connectivity values. Before computing correlation coefficients, connectivity values were corrected by regressing out physiological covariates. The green line denotes the absolute correlation coefficient value of 0.3 while the blue line denotes the absolute correlation coefficient value of 0.5.

2.2 Feature rankings by t-test and feature importance

2.2.1 The effect of LSD on functional connectivity

About 23% (1993/8646) unique correlation coefficients significantly differed across LSD and placebo conditions ($p < 0.05$). Among these connections, we observed mostly stronger FC under LSD (Figure 1A-C, main manuscript). Of the top 50 connections (ranked by t-statistic of the difference between conditions; Figure S2A), only two connections showed weaker FC under LSD, each connecting two regions in the occipital cortex: bilateral connections between occipital poles and the occipital fusiform gyri (ranked 6th and 8th respectively; Figure S2B). Six of the remaining top ten connected parietal and frontal regions (Figure S2A-B).

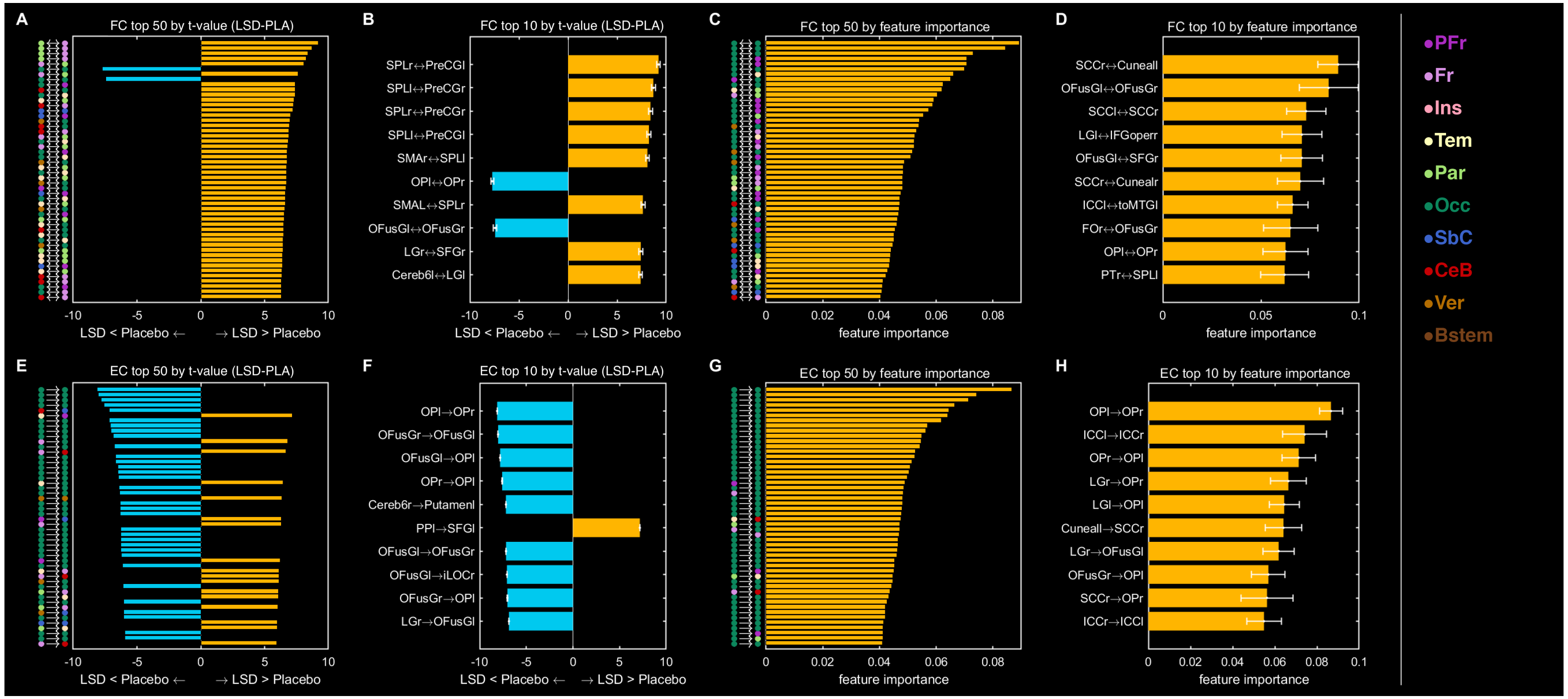

Figure S2: **Ranked differences in functional (FC) and effective connectivity (EC) between LSD and placebo conditions.** **A** Top 50 connections by across-participant t-statistic of the difference in FC between the LSD and placebo conditions. **B** Top 10 connections by across-participant t-statistic of the difference in FC between the LSD and placebo conditions. **C** Top 50 connections by feature importance estimate for the FC classification model. See section 1.6 for a detailed definition of feature importance. **D** Top 10 connections by feature importance estimate for the FC classification model. **E** Top 50 connections by across-participant t-statistic of the difference in EC between the LSD and placebo conditions. **F** Top 10 connections by across-participant t-statistic of the difference in EC between the LSD and placebo conditions. **G** Top 50 connections by feature importance estimate for the EC classification model. **H** Top 10 connections by feature importance estimate for the EC classification model. For **A,C,E,G**, coloured circles indicate the area into which each region of interest (ROI) is categorized. For **B,D,F,H**: orange and blue bars indicate increases and decreases, respectively, in connectivity in the LSD condition; abbreviations indicate the ROIs forming each connection. For **B,F**, errorbars represent the across-participant standard deviation of the differences in connectivity between conditions. For **D,H**, errorbars represent the across-fold standard deviation for the feature importance estimates. For **C,D,G,H**, please note that only the top 10 or top 50 connections have been displayed to reduce the information load for visualization. For E,F,G,H, directionality of connections is indicated using the → on the y-axes. PFr: Prefrontal cortex. Fr: Frontal cortex. Ins: Insular cortex. Tem: Temporal cortex. Par: Parietal cortex. Occ: Occipital cortex. SbC: Subcortical regions. CeB: Cerebellum. Ver: Vermis. Bstem: Brainstem. For other labels, see region of interest label abbreviation key in Section 1.9

2.2.2 The effect of LSD on effective connectivity

About 13% (2184/17424) effective connections strength coefficients significantly differed across conditions ($p < 0.05$). As with LSD-induced changes in FC, we observed primarily stronger EC under LSD (Figure 1E-G, main manuscript). However, surprisingly, among the most prominent differences was *weaker* EC between occipital brain regions under LSD, including 33 out of the top 50 (Figure S2E) and nine out of the top 10 connections (Figure S2F) ranked by t-statistic of the difference between conditions. Only 13 out of the top 50 connections did not involve an occipital region. Of the top 10 connections, only 2 were not between occipital regions. These included right cerebellum to left putamen, which showed weaker EC, whereas left planum temporale-to-left superior frontal gyrus connections showed stronger EC under LSD compared to placebo (Figure S2F).

2.2.3 The effect of LSD on inhibitory self-connections

About 30% (39/132) inhibitory self-connections significantly differed across conditions ($p < 0.05$). Occipital regions once again displayed the greatest effects showing more negative or decreased local connectivity under LSD (Figure 4, main manuscript). One exception to this pattern was an increase for the Vermis 8 self-connection, which ranked second in terms of absolute t-value. The top-ranked self-connection was the left occipital pole, which was involved in the first-, third-, fourth-, and ninth-ranked non-self-connections by absolute t-value.

2.3 Classification performances

	N_{tree}	BAC	AUC	SE	SP	PPV	NPV	p-value[a]
FC	8646	0.86	0.95	0.87	0.84	0.85	0.87	<0.001
EC	17424	0.91	0.96	0.91	0.91	0.92	0.92	<0.001

Table S3: **Classification Performances**. Performance measures for models trained on either functional connectivity (FC) or effective connectivity (EC). N_{tree}: Number of trees used in the random forest models. BAC: Balanced accuracy. AUC: Area-under-the-curve. SE: Sensitivity. SP: Specificity. PPV: positive predictive value. NPV: negative predictive value. [a] p-values for balanced accuracy were obtained through permutation test with 1000 label permutations.

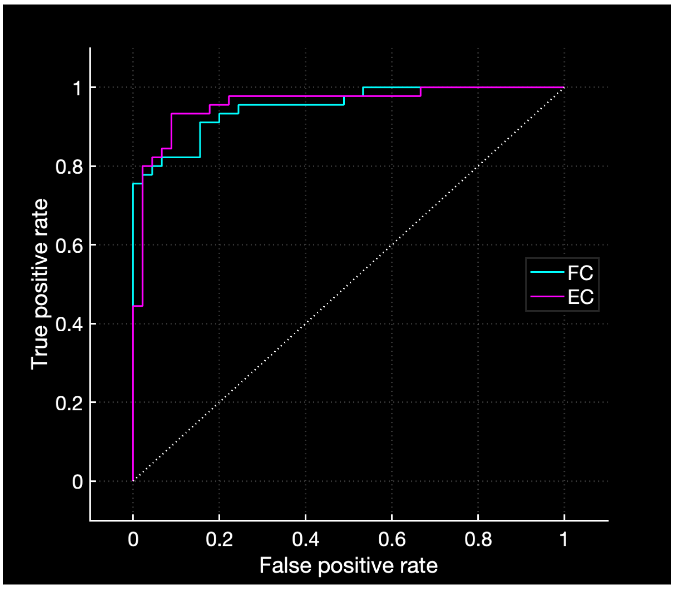

Figure S3: **Receiver Operating Characteristic curves for classification of LSD vs placebo conditions**. Shown are Receiver Operating Characteristic (**ROC**) curves for random forests trained on either effective connectivity (**EC**) features in cyan or functional connectivity (**FC**) features in magenta to classify LSD vs placebo conditions.

2.4 Behavioral PLSC results

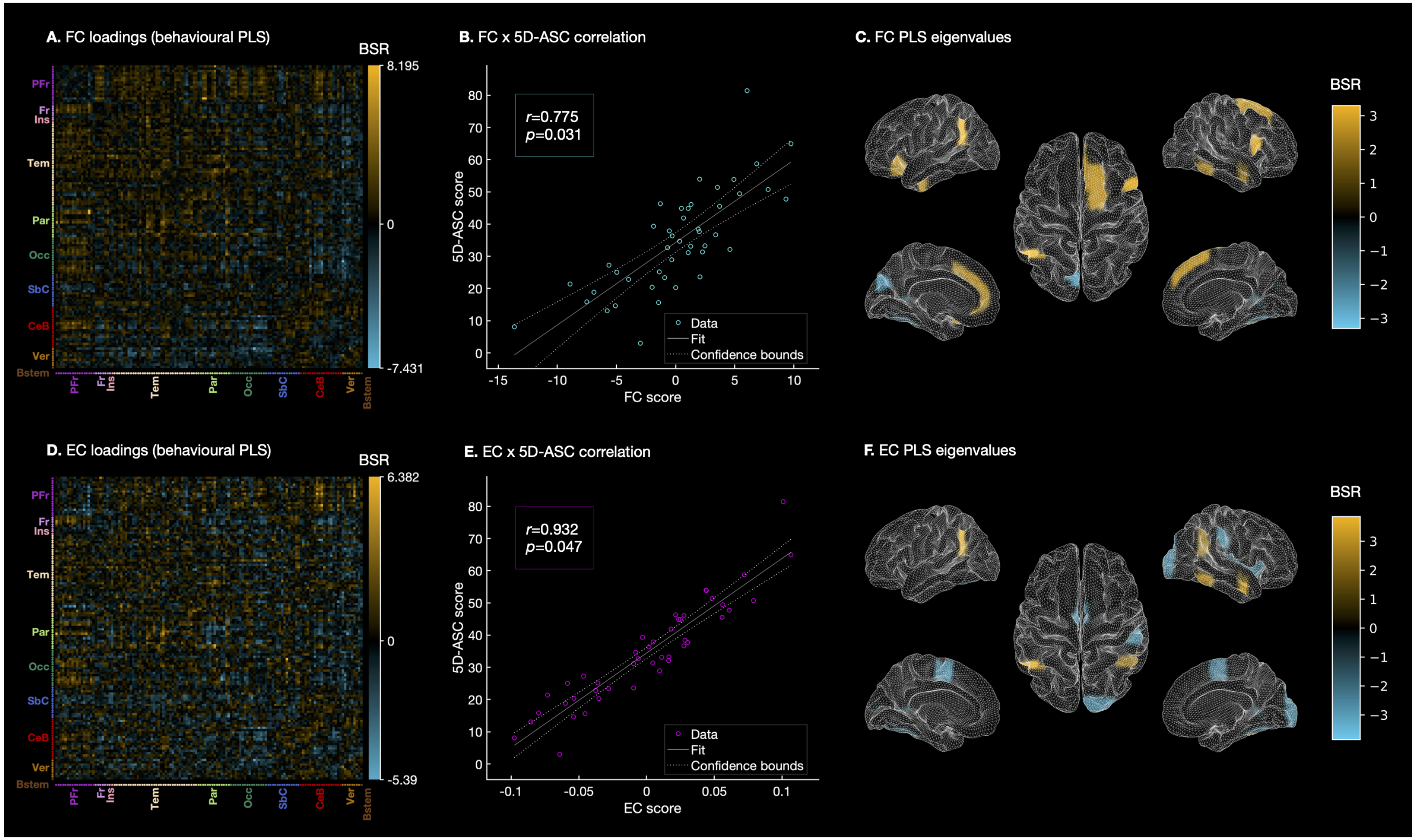

Figure S4: **Partial least squares correlation (PLSC) analysis results showing brain-behaviour relationships.** Bootstrap ratios (BSRs) for whole-brain functional connectivity (**FC**) or whole-brain effective connectivity (**EC**), which correlated with the global score of the 5 Dimensions of Altered States of Consciousness (5D-ASC) scale [28, 29], are shown in **A** or **D**, respectively. BSRs are the ratios of the loadings on the latent variable and the standard errors estimated from bootstrapping. The larger the magnitude of a BSR, the larger the weight (i.e., the loading on the latent variable) and the smaller the standard error (i.e., higher stability; [27, 30]). BSRs can be understood analogous to z-scores provided that bootstrap distributions are approximately normal [31]. Panels **B** and **E** show the correlations between 5D-ASC scores and FC or EC scores, respectively. Leading Eigenvectors highlighting areas with the strongest brain-behaviour relationships are shown in panels **C** for FC and **F** for EC. PFr: Prefrontal cortex. Fr: Frontal cortex. Ins: Insular cortex. Tem: Temporal cortex. Par: Parietal cortex. Occ: Occipital cortex. SbC: Subcortical regions. CeB: Cerebellum. Ver: Vermis. Bstem: Brainstem.

References

1. Dolder, P. C., Schmid, Y., Müller, F., Borgwardt, S. & Liechti, M. E. LSD acutely impairs fear recognition and enhances emotional empathy and sociality. *Neuropsychopharmacology* **41,** 2638–2646 (2016).
2. Müller, F., Dolder, P. C., Schmidt, A., Liechti, M. E. & Borgwardt, S. Altered network hub connectivity after acute LSD administration. *NeuroImage Clinical* **18,** 694–701 (2018).
3. Holze, F. *et al.* Distinct acute effects of LSD, MDMA and D-amphetamine in healthy subjects. *Neuropsychopharmacology* **45,** 462–471 (2020).
4. Müller, F. *et al.* Increased thalamic resting-state connectivity as a core driver of LSD-induced hallucinations. *Acta Psychiatrica Scandinavica* **136,** 648–657 (2017).
5. Whitfield-Gabrieli, S. & Nieto-Castanon, A. Conn: a functional connectivity toolbox for correlated and anticorrelated brain networks. *Brain Connectivity* **2,** 125–141 (2012).
6. Friston, K. J., Ashburner, J., Kliebel, S., Nichols, T. & Penny, W. *Statistical Parametric Mapping: The Analysis of Functional Brain Images* 1st ed. ISBN: 978-0-12-372560-8 (Elsevier, 2007).
7. Ashburner, J. & Friston, K. Unified segmentation. *NeuroImage* **26,** 839–51 (2005).
8. Birn, R. *et al.* The effect of scan length on the reliability of resting-state fMRI connectivity estimates. *NeuroImage* **83,** 550–8 (2013).
9. Power, J., Barnes, K., Snyder, A., Schlaggar, B. & Petersen, S. Spurious but systematic correlations in functional connectivity MRI networks arise from subject motion. *NeuroImage* **59,** 2142–54 (2012).
10. McCulloch, D. E.-W. *et al.* Psychedelic resting-state neuroimaging: A review and perspective on balancing replication and novel analyses. *Neuroscience & Biobehavioral Reviews* **138,** 104689 (2022).
11. Friston, K. J., Harrison, L. & Penny, W. Dynamic causal modelling. *NeuroImage* **19,** 1273–1302 (2003).
12. Buxton, R., Wong, E. & Frank, L. Dynamics of blood flow and oxygenation changes during brain activation: the balloon model. *Magnetic Resonance in Medicine.* **39,** 855–864 (1998).
13. Friston, K., Mechelli, A., Turner, R. & Price, C. Nonlinear responses in fMRI: the Balloon model, Volterra kernels and other hemodynamics. *NeuroImage* **12,** 466–477. ISSN: 1053-8119 (2000).
14. Havlicek, M. *et al.* Physiologically informed dynamic causal modeling of fMRI data. *NeuroImage* **122,** 355–372 (2015).
15. Stephan, K., Weiskopf, N., Drysdale, P., Robinson, P. & Friston, K. Comparing hemodynamic models with DCM. *NeuroImage* **38,** 387–401 (2007).

16. Frässle, S. *et al.* Regression DCM for fMRI. *NeuroImage* **155,** 406–421 (2017).

17. Frässle, S. *et al.* Regression dynamic causal modeling for resting-state fMRI. *Human Brain Mapping* **42,** 2159–2180 (2021).

18. Dawid, A. P. Statistical causality from a decision-theoretic perspective. *Annual Review of Statistics and Its Application* **2,** 273–303 (2015).

19. Breiman, L. Random Forests. *Machine Learning* **45,** 5–32 (2001).

20. Koutsouleris, N. *et al.* Disease prediction in the at-risk mental state for psychosis using neuroanatomical biomarkers: results from the FePsy study. *Schizophrenia Bulletin* **38,** 1234–46 (2012).

21. Frässle, S. *et al.* TAPAS: an open-source software package for Translational Neuromodeling and Computational Psychiatry. *Frontiers in Psychiatry* **12.** ISSN: 1664-0640 (2021).

22. Groppe, D. *fdr_bh* https://www.mathworks.com/matlabcentral/fileexchange/27418-fdr_bh), MATLAB Central File Exchange. Retrieved 08-03-2021. 2015.

23. Van Rossum, G. & Drake, F. L. *Python 3 Reference Manual* ISBN: 1441412697 (CreateSpace, Scotts Valley, CA, 2009).

24. Frässle, S. *et al.* A generative model of whole-brain effective connectivity. *NeuroImage* **179** (2018).

25. Benjamini, Y. & Hochberg, Y. Controlling the false discovery rate: A practical and powerful approach to multiple testing. *Journal of the Royal Statistical Society, Series B (Methodological)* **57,** 289–300 (1995).

26. McIntosh, A., Bookstein, F., Haxby, J. V. & Grady, C. Spatial pattern analysis of functional brain images using partial least squares. *NeuroImage* **3,** 143–157 (1996).

27. McIntosh, A. & Lobaugh, N. Partial least squares analysis of neuroimaging data: applications and advances. *NeuroImage* **23,** Suppl 1:S250–63 (2004).

28. Dittrich, A. The standardized psychometric assessment of altered states of consciousness (ASCs) in humans. *Pharmacopsychiatry* **31,** 80–84 (1998).

29. Studerus, E., Gamma, A. & Vollenweider, F. X. Psychometric evaluation of the altered states of consciousness rating scale (OAV). *PloS one* **5,** e12412 (2010).

30. Mišić, B. *et al.* Network-level structure-function relationships in human neocortex. *Cerebral Cortex* **26,** 3285–3296 (2016).

31. Efron, B. & Tibshirani, R. Bootstrap methods for standard errors, confidence intervals, and other measures of statistical accuracy. *Statistical science,* 54–75 (1986).
